# Supplementary material for: Chromothripsis during telomere crisis is independent of NHEJ, and consistent with a replicative origin
Source: Genome Res. 2019 May;29(5):737–49. doi: 10.1101/gr.240705.118 (PMC6499312; doi:10.1101/gr.240705.118)
Supplement: Supplemental Material [file supp_gr.240705.118_Supplemental_file_1.zip › contigs/annotated_contigs/DB46/contig.2.DB46_length_526_mean_cov_3.91634980989.docx]

**DB46_length_526_mean_cov_3.91634980989**

```
ATGCTACCTCTATAAAGGACCAAAATAGTATTTTTAAAAGAATTTATCAAAGTAACTATAATAAAAGTATTTAATGGTATTTGTATTGC
 >chr14:86102560-86102722 + E=7e-86
AAACATTGAAATTCTACCTGGCAGTTTAAAGAACATCACTAACATTGCCTAAATGTAGGAAAATCAAAGGT|GA|CAGAGCGAGACACT
                                                                        >chr14:86105858
GTCTCAAAAAAAAAAAGAAAAAAAAAAATAAAGAATTTCTGCTAGTGAAGCACCATGGTGCGGTGAGGGAAAACAGCCTGATTTGAAAT
-86106231 - E=7e-195
CCTAAGCCCTGTGTTTTCAAAGCTCATTCCATCACTTATTGTAAGAGCTTAGCCAGTCCTTTCTGGGGGCTTCAGTGTTACCATCTTTA

AAATGGGTAATGCACATAATAATATCAATAGTCTATAAATATAAATTCTACTAACACACACTCTAATTTTAGCCAAACTCATGAGAGAA

AATTCAGCAGCTATACAAGAAGATTGAGATAAATCAATCATTGCTACCATCATTTGGGCTCACTAAAAAGTAGCAGAAAATGG
```
